# Percutaneous cholecystostomy tube placement as a bridge to cholecystectomy for grade III acute cholecystitis: A national analysis

**DOI:** 10.1016/j.sopen.2024.01.006

**Published:** 2024-01-14

**Authors:** Joanna Curry, Nikhil Chervu, Nam Yong Cho, Joseph Hadaya, Amulya Vadlakonda, Shineui Kim, Jessica Keeley, Peyman Benharash

**Affiliations:** a Cardiovascular Outcomes Research Laboratories (CORELAB), University of California Los Angeles, California, USA; b Department of Surgery, David Geffen School of Medicine at UCLA, University of California Los Angeles, California, USA; c Department of Surgery, Harbor-UCLA Medical Center, Torrance, CA, USA

**Keywords:** Percutaneous cholecystostomy, Cholecystectomy, Outcomes, Resource utilization

## Abstract

**Introduction:**

Percutaneous cholecystostomy (PCT) is an alternative to cholecystectomy (CCY) in high-risk surgical candidates with severe acute cholecystitis. A subset of these patients ultimately undergo delayed CCY. We therefore compared outcomes of delayed CCY in patients with grade III acute cholecystitis who received a PCT on index admission, to those who did not.

**Methods:**

Non-elective adult hospitalizations for grade III acute cholecystitis that underwent delayed CCY were identified in the 2016–2020 Nationwide Readmission Database. Patients who received a PCT during their index admission comprised the *PCT* group (others: *Non-PCT)*. Outcomes were assessed for the CCY hospitalization. Entropy balancing was used to generate sample weights to adjust for differences in baseline characteristics. Regression models were created to evaluate the association between *PCT* and the outcomes of interest.

**Results:**

Of an estimated 13,782 patients, 13.3 % comprised *PCT*. Compared to *Non-PCT*, *PCT* were older (71.1 ± 13.1 vs 67.4 ± 15.3 years) and more commonly in the highest income quartile (22.5 vs 16.1 %, both *p* < 0.001). After risk adjustment, *PCT* was associated with reduced odds of respiratory (AOR 0.67, CI 0.54–0.83) and infectious (AOR 0.77, CI 0.62–0.96) complications after eventual CCY. Finally, *PCT* had comparable pLOS (β +0.31, CI [−0.14, 0.77]) and operative hospitalization costs (β $800, CI [−2300, +600]).

**Conclusion:**

In the present study, *PCT* was associated with decreased odds of perioperative complications and comparable resource utilization upon readmission CCY. Our findings suggest that PCT may be helpful in bridging patients with grade III acute cholecystitis to eventual CCY.

## Introduction

Cholelithiasis afflicts >20 million people in the United States and accounts for ∼$5 billion in healthcare expenditures, annually. Importantly, an estimated 1 % of patients with cholelithiasis will develop acute cholecystitis (AC) requiring hospitalization [1,2]. Cholecystectomy (CCY) remains the definitive treatment for AC, as patients may otherwise experience recurrence and disease progression beyond their initial presentation [3]. However, contemporary management guidelines have endorsed the use of percutaneous cholecystostomy (PCT) or definitive antibiotic therapy in select patients who may be at increased perioperative risk for cholecystectomy [4]. While this approach may obviate the need for urgent surgery, PCT may, in fact, complicate future cholecystectomy due to increased risk of bile duct injury [5].

The use of PCT as a bridge to future CCY has shown mixed results in single center studies, partly due to the heterogeneity in study populations and small sample size [[6], [7], [8]]. The 2018 Tokyo Guidelines for the treatment of gallbladder and biliary pathology classifies cholecystitis into grade I-III, with grade III defined as acute pathology with additional organ dysfunction. In those with grade III disease who may be at increased risk for surgery, the guidelines have recommended initial percutaneous biliary drainage followed by delayed CCY [4,9]. Nevertheless, outcomes between nonoperatively managed patients who received PCT or definitive antibiotic therapy, rather than CCY, during their index admission for cholecystitis remain ill-defined. Given practice variability, additional research is required to examine the impact of prior PCT on outcomes of CCY deferred to a later date [10].

The present study sought to examine the association of initial PCT placement on perioperative outcomes and resource utilization of delayed CCY. We hypothesized that index PCT would be associated with shorter time to CCY, higher odds of perioperative complications, as well as increased postoperative length of stay (pLOS) and hospitalization costs.

## Methods

We performed a retrospective cohort study using the 2016 to 2020 Nationwide Readmissions Database (NRD). Maintained by the Healthcare Cost and Utilization Project, the NRD is the largest readmissions database providing accurate estimates for 60 % of annual US hospitalizations [11]. The NRD uses unique hospital and patient identifiers, facilitating analyses across multiple hospitalizations within the calendar year.

**Fig. 1:**
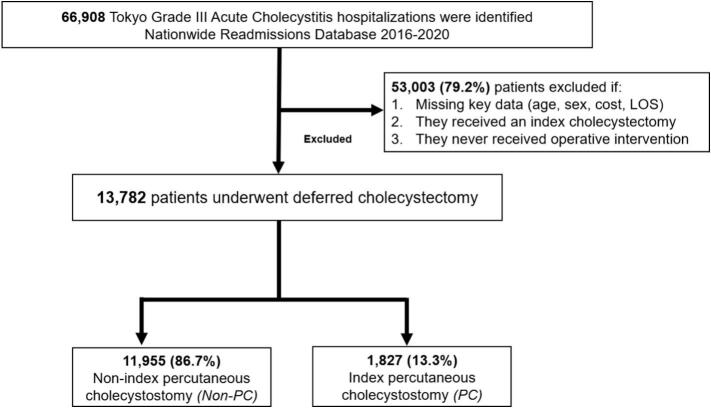
Exclusion criteria.

All non-elective adult (≥18 years) hospitalizations with a primary diagnosis of acute cholecystitis were identified using relevant *International Classification of Diseases*, *Tenth Revision* (ICD-10) codes (Supplemental Table 1). Patients who underwent CCY at the index hospitalization were excluded from study. Grade III AC was defined as previously described by Dimou et al. [12]. Given the structure of NRD, only those who underwent CCY within the same calendar year of their initial admission were included for analysis. Patients with a diagnosis of choledocholithiasis, gallstone pancreatitis, or cholangitis were excluded. This was done to minimize the influence of outliers and complex cases that may be subject to more nuanced decision-making that may not be adequately represented within the NRD. Records with missing data for age, sex, costs, and length of stay, were also excluded (0.9 %; Fig. 1). Patients were then stratified into *PCT* and *Non-PCT* on the basis of whether they received a PCT during the index hospitalization.

Patient and hospital characteristics including age, sex, income quartile, primary payer, hospital setting, teaching status, and bed size were defined according to the NRD data dictionary [11]. Additionally, perioperative complications were determined using previously published ICD-10 codes [13]. The van Walraven modification of the Elixhauser Comorbidity Index was utilized to evaluate the burden of chronic comorbid conditions [14,15]. Hospitalization costs were computed using institution-specific cost-to-charge ratios, which was then adjusted for inflation to the 2020 Personal Health Index [11,16].

The primary outcomes of interest were perioperative complications. Secondary outcomes included pLOS, hospitalization costs, and rates of non-home discharge. All outcomes were assessed for the CCY hospitalization in order to ensure appropriate postoperative comparison between groups. Perioperative outcomes included neurologic (stroke or transient ischemic attack), cardiac (cardiac arrest or myocardial infarction), respiratory (acute respiratory failure, prolonged ventilation, pneumothorax, or acute respiratory distress syndrome), gastrointestinal or hepatobiliary (bile leak, upper gastrointestinal bleeding, iatrogenic bowel perforation, liver infarction, and hepatic vein thrombosis), thrombotic (deep vein thrombosis and pulmonary embolism), and infectious (urinary tract infection, sepsis, wound infection) complications.

**Supplemental Fig. 1:**
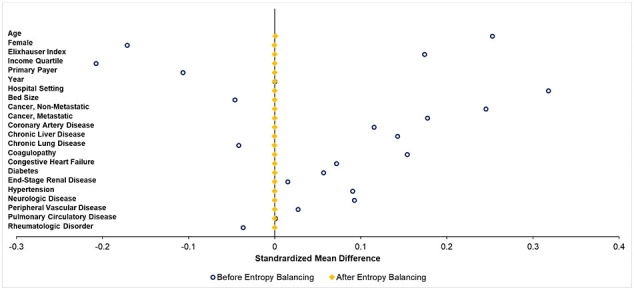
Pre- and post-covariate balancing after entropy balancing.

Categorical data are reported as group proportions (%) and continuous data as means with standard deviation (SD) or medians with interquartile ranges (IQR). Adjusted Wald and Pearson's χ^2^ tests were used to determine the significance of intergroup differences for continuous and categorical variables, respectively. The significance of temporal trends were evaluated using Cuzick's non-parametric rank-based test (*nptrend*) [17]. A multivariable logistic regression model was constructed to examine factors associated with PCT. Prior to examining perioperative outcomes and resource utilization, entropy balancing was used to obtain optimal sample weights to balance covariates between groups (Supplemental Fig. 1). Unlike propensity score matching, this methodology maintains the entire cohort for analysis and reduces sampling bias [[18], [19], [20]]. Logistic and linear regression models using entropy balanced sample weights were then utilized to determine the association of PCT with the aforementioned outcomes. All models were optimized using Bayesian information criteria and receiver operating characteristics (C-statistic) [21]. Regression outputs are reported as adjusted odds ratios (AOR) or beta-coefficients (β) with 95 % confidence intervals (CI). An α <0.05 was set for significance.

All statistical analyses were performed using Stata 16.1 software (StataCorp, College Station, TX). This study was deemed exempt from full review by the Institutional Review Board at the University of California, Los Angeles.

## Results

**Fig. 2:**
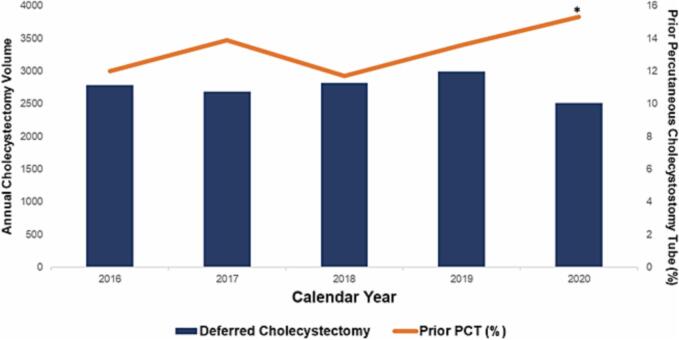
National trends in overall volume of cholecystectomy and the proportion of cholecystectomies that are Non-Index, 2016–2020. *nptrend* < 0.001.

Of an estimated 13,782 patients who met study criteria, 1827 (13.3 %) received a PCT at the index hospitalization for grade III acute cholecystitis, while others were initially managed medically. Throughout the study period, the proportion of patients having an index PCT and receiving a delayed CCY increased from 12.0 to 15.3 % (*nptrend* = 0.02; Fig. 2). Among the grade III AC group, 25.5 % of patients who received an index PCT underwent delayed CCY, while 33.2 % of those who did not required a subsequent readmission CCY. Compared to *Non-PCT*, *PCT* were older (71.1 ± 13.1 vs 67.4 ± 15.3 years, *p* < 0.001), less commonly female (34.1 vs 43.6 %, p < 0.001), and had a higher burden of comorbid disease as measured by the Elixhauser Index (5 [3–6] vs 4 [3–6], *p* < 0.001). Additionally, *PCT* were more likely to be in the highest income quartile (22.5 vs 16.1 %*, p* < 0.001), less commonly uninsured (1.6 vs 2.8 %, *p* = 0.002), and more frequently presented to metropolitan teaching hospitals (79.5 vs 64.6 %, *p* < 0.001; Table 1). Finally, prior PCT was associated with a shorter time to CCY readmission (51 ± 42 vs 72 ± 74 days, *p* < 0.001; Table 1).

**Table 1:** Demographic and hospital characteristics of patients who received a percutaneous cholecystostomy during index hospitalization (PCT) and those who did not (Non-PCT) from 2016 to 2020; SD, standard deviation; IQR, interquartile range.

	Non-PCT (n = 11,955)	PCT (n = 1827)	p-Value
Age (years ± SD)	67.4 ± 15.3	71.1 ± 13.1	<0.001
Female (%)	43.6	34.1	<0.001
Elixhauser Comorbidity Index (median [IQR])	4 [3–6]	5 [3–6]	<0.001
Income quartile (%)			<0.001
>75 %	16.1	22.5	
51–75 %	22.5	27.0	
26–50 %	29.1	24.7	
0–25 %	30.8	24.8	
Insurance coverage (%)			0.002
Private	14.9	15.1	
Medicare	68.1	73.1	
Medicaid	10.8	8.7	
Uninsured	2.8	1.6	
Hospital teaching status (%)			<0.001
Non-metropolitan	9.6	2.5	
Metropolitan non-teaching	25.7	18.0	
Metropolitan teaching	64.6	79.5	
Bed size (%)			<0.001
Large	53.5	55.6	
Medium	28.6	27.5	
Small	17.9	16.9	
Comorbidities (%)			
Cancer, non-metastatic	3.9	4.9	0.15
Cancer, metastatic	1.7	1.3	0.32
Cardiac arrhythmia	34.0	39.1	0.003
Chronic liver disease	8.0	11.5	0.002
Chronic lung disease	28.1	25.4	0.09
Coagulopathy	11.1	16.5	<0.001
Congestive heart failure	24.7	27.8	0.05
Diabetes	44.4	47.3	0.09
End-stage renal disease	34.2	34.9	0.68
Hypertension	78.9	82.6	0.01
Neurologic disorder	10.9	13.5	0.02
Obesity	23.8	28.4	0.004
Peripheral vascular disease	11.8	12.9	0.39
Pulmonary circulatory disease	7.0	7.8	0.41
Rheumatologic disorder	4.2	3.5	0.29

Following risk adjustment, increasing age (Adjusted Odds Ratio [AOR] 1.02/year, 95 % Confidence Interval [CI] 1.01–1.03, p < 0.001) and Elixhauser Index (AOR 1.14/unit, CI 1.08–1.21, p < 0.001) were associated with greater odds of receiving a PCT, while female sex (AOR 0.70, CI 0.60–0.82, p < 0.001) was linked with reduced odds of receiving index PCT. A full list of factors associated with receipt of PCT can be found in Table 2.

**Table 2:** Factors associated with index PCT among patients with grade III cholecystitis who undergo delayed cholecystectomy. AOR, adjusted odds ratio; CI, confidence interval; IQR, interquartile range.

	AOR	95 % CI		p-Value
Age	1.02	1.01	1.03	<0.001
Female	0.70	0.60	0.82	<0.001
Elixhauser Comorbidity Index (median [IQR])	1.14	1.08	1.21	<0.001
Heart failure	0.94	0.78	1.14	0.66
Chronic lung disease	0.76	0.63	0.91	0.007
Coagulopathy	1.22	0.98	1.52	0.07
Hospital teaching status				<0.001
Non-metropolitan hospital	Reference			
Metropolitan non-teaching	2.36	1.46	3.80	<0.001
Metropolitan hospital	4.21	2.65	6.68	<0.001
Income quartile				
0–25th percentile	Reference			
26th to 50th percentile	0.96	0.79	1.18	0.71
51st to 75th percentile	1.26	1.01	1.56	0.04
76 to 100th percentile	1.37	1.10	1.69	0.004

On unadjusted analysis, *PCT* had lower rates of respiratory (13.3 vs 18.2 %, *p* = 0.001), infectious (11.3 vs 14.3 %, *p* = 0.02), and gastrointestinal complications (2.5 vs 4.4 %, *p* = 0.01) compared to *non-PCT*. Furthermore, *PCT* had comparable pLOS (4 [2–7] vs 4 [2–6] days, *p* = 0.10), hospitalization costs ($17,300 [11,700 - 26,700] vs $17,700 [12,900 - 26,200], *p* = 0.99), and a similar incidence of nonhome discharge (19.5 vs 21.8 %, *p* = 0.12).

**Fig. 3:**
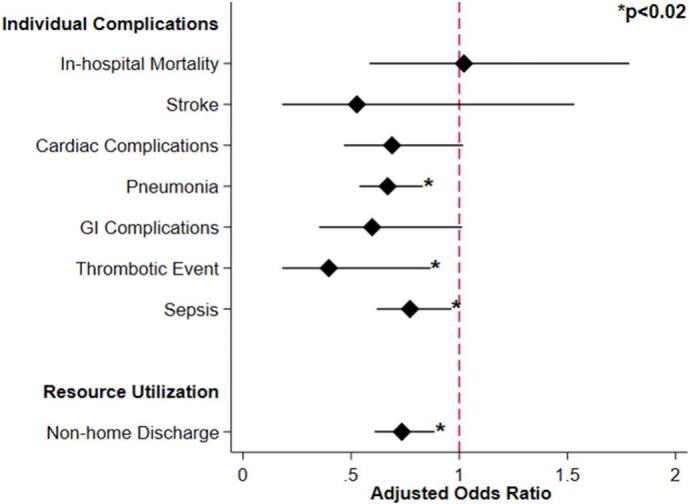
Association of percutaneous cholecystectomy tube with outcomes of those undergoing delayed cholecystectomy (Reference = *Non-PC*).

After entropy balancing, PCT was associated with reduced odds respiratory (AOR 0.67, CI 0.54–0.83, *p* < 0.001), thrombotic (AOR 0.40, CI [0.18–0.87], *p* = 0.02), and infectious complications (AOR 0.77, CI 0.62–0.96, p < 0.001; Fig. 3). Additionally, PCT was linked with similar odds of gastrointestinal complications (AOR 0.60, CI 0.35–1.01, *p* = 0.06; Fig. 3). Finally, PCT was associated with comparable pLOS (β +0.31, CI [−0.14, +0.77], *p* < 0.18) and operative hospitalization costs (β -$800, 95%CI [−2300, +600], *p* < 0.27), as well as lower odds of non-home discharge (AOR 0.73, CI 0.61–0.89, *p* = 0.001; Table 3) compared to non-PCT.

**Table 3:** Risk-adjusted outcomes following delayed cholecystectomy for patients with a history of cholecystitis who underwent initial cholecystectomy tube placement relative to those who did not. Outcomes reported as adjusted odds ratio or beta coefficient (reference group: Non-PCT). AOR, adjusted odds ratio; β, beta coefficient; CI, confidence interval; CCY, Cholecystectomy; GI, gastrointestinal; pLOS, postoperative length of stay.

	AOR or β Coefficient	95 % CI	p-Value
Resource utilization			
CCY hospitalization cost (USD $1000) [IQR]	+800	[−2300 − +600]	0.27
pLOS (days) [IQR]	0.31	[−0.14 − +0.77]	0.18
Non-home discharge	0.73	[0.61–0.89]	<0.001
Complications			
Neurological^a^	0.53	[0.18–1.53]	0.24
Cardiac^b^	0.69	[0.47–1.02]	0.06
Respiratory^c^	0.67	[0.54–0.83]	<0.001
GI/hepatobiliary^d^	0.60	[0.35–1.01]	0.06
Thrombotic	0.40	[0.18–0.87]	0.02
Sepsis	0.77	[0.62–0.96]	0.02

a Stroke or transient ischemic attack.

b Cardiac arrest or myocardial infarction.

c Acute respiratory failure, prolonged mechanical ventilation, or acute respiratory distress syndrome.

d Bile leak, upper gastrointestinal bleeding, iatrogenic bowel perforation, liver infarction, and hepatic vein thrombosis.

## Discussion

Nonoperative management of AC has been increasingly employed in recent years, with the COVID-19 pandemic further altering surgical decision-making [22,23]. In this nationwide retrospective study, we observed a significant increase in the utilization of PCT as a bridge to CCY among those managed nonoperatively at the index hospitalization for grade III AC. Factors associated with receipt of PCT included increased age, sex, and burden of comorbid disease. Receiving an index PCT was associated with lower odds of experiencing perioperative complications at the time of delayed CCY. Furthermore, index PCT was associated with comparable pLOS and operative hospitalization costs, as well as decreased odds of experiencing non-home discharge. Several of these findings warrant further discussion.

Prior research has provided evidence for the use of PCT in high-risk surgical populations as either definitive treatment for cholecystitis or as a bridge to CCY [24,25]. Although PCT is one non-operative modality to treat AC, others have suggested that bowel rest and definitive antibiotics may be sufficient for patients with AC who are felt to be poor surgical candidates at initial presentation [26]. In the present analysis, 25.5 % of patients who received a PCT underwent delayed inpatient CCY, compared to the 33 % delayed inpatient CCY rate observed among those managed with antibiotics and supportive care. This is in congruence with a single-center retrospective study of 245 patients who received PCT for cholecystitis, where 29 % of patients went on to require interval CCY [27]. Additionally, Suzuki et al. observed that among patients receiving antibiotic treatment for initial presentation of AC, 43 % of patients readmitted for reoccurrence required urgent CCY [28]. While the need for delayed surgery is well established, current literature suggests that deferred CCY is associated with increased risk of perioperative complications and wound infections compared to those who received CCY on initial presentation [29,30]. However, patients who receive nonoperative management during the index admission often have more severe gallbladder inflammation and a higher burden of comorbid disease [31]. Given this, the two cohorts are difficult to compare, and it is imperative to delineate how different non-operative treatment options impact delayed perioperative outcomes within this distinct population. Therefore, the increasing utilization of index PCT as a bridge to deferred CCY warrants further discussion due to its potential impact on clinical outcomes and expenditures.

Given the significant recurrent rate of AC after cholecystostomy, prior literature has recommended its use only as a bridge to eventual elective CCY. In our analysis, which was limited to solely patients who were managed non-operatively at the initial hospitalization for AC, rates of PCT use for Tokyo grade III increased from 12.0 to 15.3 % from 2016 to 2020. Despite this rise, however, only a small fraction of the cohort received a PCT, despite guidelines suggesting it should be strongly considered if CCY will be delayed beyond the initial hospitalization. This may be related to lack of access to PCT or concerns that PCT tubes may delay eventual CCY. On the contrary, our data suggests that, in severe AC that is managed non-operatively, PCT appears to be associated with superior outcomes compared to antibiotics alone. The present study demonstrates that PCT followed by deferred CCY is increasingly more common in an acutely ill population and further establishes potential clinical benefit within this distinct cohort. Given the unique pathology and high rate of disease recurrence within this vulnerable population, our work emphasizes the importance of index care choices on outcomes of delayed CCY.

In our analysis, index PCT was associated with decreased odds of respiratory, thrombotic, and infectious complications at the surgical hospitalization for CCY. There are several plausible explanations for these findings. Given the benefit of continual drainage and potential symptomatic benefit from PCT, patients who received a PCT for cholecystitis may have lower rates of recurrent cholecystitis while the tube is still in place. Indeed, we noted non-elective admissions for definitive CCY to be greater among those who did not initially receive a PCT. The use of PCT has additionally been associated with reduced systemic inflammatory response and less chronic inflammation of the gallbladder, which may facilitate later CCY [32,33]. Alternatively, patients who received PCT may have been fit enough to tolerate PCT placement, though this can often be performed under local anesthesia [34]. Clinical endpoints aside, we found that those bridged with PCT had comparable pLOS and hospitalization costs, as well as reduced odds of nonhome discharge. While prior research has found PCT to be associated with increased cumulative care costs compared to index CCY, the present analysis demonstrates no increase when we look only among those initially managed nonoperatively [35,36]. The findings of the present study demonstrate that index PCT is associated with superior clinical outcomes and comparable resource utilization at deferred CCY. These findings should be considered when evaluating recommendations for value-based care practices.

Our study has several limitations related to its design and the structure of the NRD. The NRD does not contain data regarding specific imaging findings or laboratory reports, though these data points are abstracted into diagnoses at each hospital. Additionally, given the retrospective design, we are unable to make causal associations between index PCT and the observed outcomes during deferred CCY. Moreover, outpatient or emergency room visits for management of cholecystostomy tubes were unable to be studied, and likely influence costs. Furthermore, NRD tracks admissions within one calendar year, and is as such unable to track patients who ultimately received a CCY in a subsequent year. In addition, patient-reported outcomes were not able to be studied, as PCT may influence quality of life. Notwithstanding, this study utilized the largest all-payer database and comprehensive statistical analysis to decrease bias and enhance the generalizability of our findings.

## Conclusion

Our findings suggest that percutaneous cholecystostomy tubes are increasingly utilized among patients undergoing non-operative management of severe acute cholecystitis. Compared to antibiotics alone, PCT was associated with lower adjusted odds of respiratory, thrombotic, and infectious complications in patients receiving deferred CCY. Finally, index PCT was associated with comparable postoperative length of stay and operative hospitalization costs, as well as decreased odds of non-home discharge. In conclusion, PCT as a bridge to cholecystectomy for grade III AC is safe, does not delay cholecystectomy, and may be associated with reduced complications and resource use compared to management without PCT.

The following are the supplementary data related to this article.

Supplemental Table 1 International Classification of Diseases, 10th Edition, Clinical-Modification Diagnosis Codes to Identify Grade III Cholecystitis, Cholecystectomy, and Cholecystostomy. Supplemental Table 1

## Funding/financial support

No external financial support was received from any source.

## Ethics approval

This study was deemed exempt from full review by the Institutional Review Board at the University of California, Los Angeles.

## CRediT authorship contribution statement

**Joanna Curry:** Conceptualization, Methodology, Formal analysis, Resources, Writing – original draft, Writing – review & editing. **Nikhil Chervu:** Conceptualization, Methodology, Formal analysis, Writing – review & editing. **Nam Yong Cho:** Conceptualization, Methodology, Writing – review & editing. **Joseph Hadaya:** Writing – review & editing. **Amulya Vadlakonda:** Methodology, Writing – review & editing. **Shineui Kim:** Methodology, Writing – review & editing. **Jessica Keeley:** Conceptualization. **Peyman Benharash:** Writing – review & editing, Supervision.

## Declaration of competing interest

The authors of this manuscript have no related conflicts of interest to declare.
