# Supplementary material for: Percutaneous cholecystostomy tube placement as a bridge to cholecystectomy for grade III acute cholecystitis: A national analysis
Source: Surg Open Sci. 2024 Jan 14;18:6–10. doi: 10.1016/j.sopen.2024.01.006 (PMC10831282; doi:10.1016/j.sopen.2024.01.006)
Supplement: Supplemental Table 1 — International Classification of Diseases, 10th Edition, Clinical-Modification Diagnosis Codes to Identify Grade III Cholecystitis, Cholecystectomy, and Cholecystostomy. [file mmc2.docx]

**Supplemental Materials:**

**Supplemental Table 1.** International Classification of Diseases, 10^th^ Edition, Clinical-Modification Diagnosis Codes to Identify Grade III Cholecystitis, Cholecystectomy, and Cholecystostomy.

|  | **ICD-10-CM/PCS Codes** |
| --- | --- |
| Acute Cholecystitis | K81.X, K80.0X, K80.12X, K80.13X, K80.18X, K80.19X |
| *Cholecystectomy* |  |
| Laparoscopic | 0FT44ZZ |
| Open | 0FT40ZZ |
| Percutaneous Cholecystostomy | 0F9430Z |
| *Tokyo Grade 3* |  |
| Hypotension | I95.x |
| Acute respiratory failure | J96.x |
| Altered mental status | R40.x |
| Renal failure | N17.x, N19.x |
| Acute hepatic dysfunction | K72.0x |
| Thrombocytopenia | D96.5x, D69.6x |

**Supplemental Figure 1.** Pre- and post-covariate balancing after entropy balancing
